# Combined treatment with dexamethasone and raloxifene totally abrogates osteoporosis and joint destruction in experimental postmenopausal arthritis

**DOI:** 10.1186/ar3371

**Published:** 2011-06-20

**Authors:** Ulrika Islander, Caroline Jochems, Alexandra Stubelius, Annica Andersson, Marie K Lagerquist, Claes Ohlsson, Hans Carlsten

**Affiliations:** 1 Centre for Bone and Arthritis Research (CBAR), The Sahlgrenska Academy, University of Gothenburg, Box 480, 405 30 Gothenburg, Sweden

**Keywords:** Raloxifene, Estradiol, Dexamethasone, collagen-induced arthritis, bone mineral density

## Abstract

**Introduction:**

Postmenopausal patients with rheumatoid arthritis (RA) are often treated with corticosteroids. Loss of estrogen, the inflammatory disease and exposure to corticosteroids all contribute to the development of osteoporosis. Therefore, our aim was to investigate if addition of the selective estrogen receptor modulator raloxifene, or estradiol, could prevent loss of bone mineral density in ovariectomized and dexamethasone treated mice with collagen-induced arthritis (CIA).

**Methods:**

Female DBA/1-mice were ovariectomized or sham-operated, and CIA was induced. Treatment with dexamethasone (Dex) (125 μg/d), estradiol (E2) (1 μg/d) or raloxifene (Ral) (120 μg/day) alone, or the combination of Dex + E2 or Dex + Ral, was started after disease onset, and continued until termination of the experiments. Arthritic paws were collected for histology and one of the femoral bones was used for measurement of bone mineral density.

**Results:**

Dex-treatment alone protected against arthritis and joint destruction, but had no effect on osteoporosis in CIA. However, additional treatment with either Ral or E2 resulted in completely preserved bone mineral density.

**Conclusions:**

Addition of raloxifene or estradiol to dexamethasone-treatment in experimental postmenopausal polyarthritis prevents generalized bone loss.

## Introduction

Rheumatoid arthritis (RA) is a progressive systemic autoimmune disease with a prevalence of about 0.5 to 1% [1] and it is characterized by symmetrical polyarthritis. Several findings indicate the involvement of sex hormones in RA. For example, the female to male incidence ratio is 4 to 5:1 before 50 years of age, and 2:1 for patients with a later onset [1,2], and the peak incidence in women coincides with the onset of menopause [3]. Chronic inflammation leads to destruction of joint cartilage and periarticular bone, as well as the development of generalized bone loss. The prevalence of osteoporosis is more than 50% in postmenopausal patients with RA [4,5]. Glucocorticoid treatment is often used to suppress inflammation in autoimmune diseases [6], but unfortunately prolonged use of glucocorticoids is associated with the development of osteoporosis and increased risk of fractures [7]. Hormone replacement therapy (HRT) is used to treat postmenopausal osteoporosis, and to compensate for the loss of natural hormones, but it is no longer recommended for long-term therapy due to the risk of serious side effects. However, HRT has also been shown to ameliorate RA, with decreased joint destruction, reduced inflammation, increased bone mineral density (BMD), and better patient health assessment [8]. Raloxifene (Ral) is a selective estrogen receptor modulator (SERM) approved for the treatment of patients with postmenopausal osteoporosis [9], and in the USA it is also approved as prophylaxis for invasive breast cancer [10]. We have previously shown that treatment with Ral or 17β-estradiol (E2) results in a reduced frequency of collagen-induced arthritis (CIA), suppressed disease severity, preserved joint histology, and maintained BMD [11]. These effects are also seen during long-term treatment, when therapy is started in patients with already established disease [12].

In this study we investigated the effects of combined treatment with Ral and glucocorticoids, or estradiol and glucocorticoids, on the development of arthritis and osteoporosis. CIA is a well-established animal model resembling RA [13]. CIA was induced in female mice that were either sham-operated or had been ovariectomized in order to assimilate a postmenopausal status in humans. We have previously shown decreased trabecular BMD in arthritic OVX mice, compared with arthritic mice with preserved endogenous estrogen production [14]. In this study, we found that treatment with the corticosteroid dexamethasone (Dex) protects against joint destruction and ameliorates the clinical signs of arthritis, although it does not prevent bone loss. However, the addition of Ral or E2 to the Dex treatment results in completely preserved BMD and protects from osteoporosis.

## Materials and methods

### Mice

The ethical committee for animal experiments at Gothenburg University approved this study. Female DBA/1 mice (TaconicM&B A/S, Ry, Denmark) were electronically tagged and kept, 5 to 10 animals per cage, under standard environmental conditions, and fed standard laboratory chow and tap water *ad libitum*.

### Ovariectomy

Ovarietomy (OVX) and sham operation were performed at 9 to 10 weeks of age. Ovaries were removed through a midline incision of the skin, and flank incisions of the peritoneum. The skin incision was then closed with metallic clips. Sham-operated animals had their ovaries exposed but not removed. Surgery was performed after the mice were anesthetized with ketamine (PfizerAB, Täby, Sweden) and medetomidin (OrionPharma, Espoo, Finland), or during Isofluran inhalation (Isofluran Baxter, Baxter Medical AB, Kista, Sweden). Carprofen (OrionPharma, Sollentuna, Sweden) was used as post-operative pain relief.

### Induction and evaluation of arthritis

One to two weeks after surgery the mice were immunized with 100 μg chicken collagen type II (CII) (SigmaAldrich, St Louis, MO, USA) dissolved in 0.1 M acetic acid and emulsified with an equal volume of incomplete Freund's adjuvant (SigmaAldrich, St Louis, MO, USA) supplemented with 0.5 mg/ml *Mycobacterium tuberculosis *(SigmaAldrich, St Louis, MO, USA). A total volume of 100 μl was injected subcutaneously at the base of the tail. Twenty one days after the first immunization, mice received a booster injection with CII emulsified in incomplete Freund's adjuvant. The animals were evaluated every two to three days for frequency and severity of arthritis until termination of the experiments. Arthritis was considered present when signs of arthritis were identified in one joint for two consecutive assessments, or in more than one joint. Scoring was performed in a blinded way without knowledge of the previous scores. Severity was graded as described previously [15], scoring 1 to 3 in each paw (maximum of 12 points per mouse) as follows: 1, swelling or erythema in one joint; 2, swelling or erythema in two joints; 3, severe swelling of the entire paw or ankylosis.

### Hormones and treatment

Mice were given intraperitoneal (ip) injections five days per week of the synthetic corticosteroid Dex (Oradexon^®^, Organon, Gothenburg, Sweden) (125 μg/mouse/day) dissolved in 0.9% sodium chloride (NaCl), subcutaneous (sc) injections of E2 (SigmaAldrich, St Louis, MO, USA) (1.0 μg/mouse/day) dissolved in Miglyol 812 (OmyaPeralta GmbH, Hamburg, Germany), or sc injections of Ral (SigmaAldrich, St Louis, MO, USA) (120 μg/mouse/day) dissolved in Miglyol 812. Control mice received ip and sc injections of 0.9% NaCl (100 μl/mouse/day) and Miglyol 812 (100 μl/mouse/day), respectively. Treatment with Dex, E2, Ral, or vehicle was started when the first mice had started to develop arthritis (day 22 to 23), and continued until termination of the experiments (day 44 to 49).

### Tissue collection and histologic examination

At the termination of the experiments, mice were anesthetized for blood withdrawal, and then euthanized by cervical dislocation. Sera were individually collected and stored at -20°C until used. Successful removal of the ovaries at the castration procedure was confirmed by weighing the uteri. One femur was placed in 70% ethanol for analysis of BMD, and the other was used for flow cytometry of bone marrow cells. Paws were placed in 4% paraformaldehyde, decalcified, and embedded in paraffin. Sections were stained with H&E and encoded before examination. In sections from each animal, the proximal and distal parts of all four paws were graded separately on a scale of 0 to 4, with the score then divided by two, which yielded a maximum histologic destruction score of 16 points per mouse, assessed as follows: 1 = synovial hypertrophy, 2 = pannus, erosions of cartilage, 3 = erosions of bone, 4 = complete ankylosis.

### Assessment of bone mineral density

One femur was subjected to a peripheral quantitative computed tomography (pQCT) scan with a Stratec pQCT XCT Research M, software version 5.4B (Norland, Fort Atkinson, WI) at a resolution of 70 μm, as described previously [16]. Trabecular BMD was determined with a metaphyseal scan at a point 3% of the length of the femur from the growth plate. The inner 45% of the area was defined as the trabecular bone compartment.

### Serologic markers of cartilage and bone remodelling

For measurement of cartilage destruction, serum levels of cartilage oligomeric matrix protein (COMP) were determined with an Animal COMP^® ^ELISA kit (AnaMar Medical AB, Uppsala, Sweden) according to the manufacturer's instructions. Bone resorption was assessed by serum levels of type I collagen fragments using the RatLaps ELISA kit (Nordic Bioscience Diagnostics, Herlev, Denmark) according to the manufacturer's instructions. The detection limits for COMP and RatLaps were 2 U/L and 6 ng/ml, respectively.

### Serologic analyses of anti-CII antibodies and IL-6

For quantification of serum IgG CII antibodies, 96-well plates (Nunc, Roskilde, Denmark) were coated overnight at 4°C with 1 μg/ml of native CII (SigmaAldrich, St Louis, MO, USA). Plates were blocked for one hour at room temperature using 0.5% BSA in PBS. Samples were diluted in 0.5% BSA-PBS, added to the plates and incubated for two hours at room temperature. Biotinylated F(ab')2 fragments of goat anti-mouse IgG (Jackson ImmunoResearch Laboratories, Suffolk, UK) was used as secondary antibody. Development was performed using extravidin peroxidase (SigmaAldrich, St Louis, MO, USA) and the enzyme substrate 3,3',5,5'-Tetramethylbenzidine (TMB) (SigmaAldrich, St Louis, MO, USA). The reaction was stopped using 1 M H_2_SO_4 _and the absorbance was measured at 405 nm in a SPECTRAmax spectrophotometer. Serum levels of IL-6 were measured using a BD™ CBA mouse inflammation kit (BD Biosciences, San Jose, CA, USA) according to the manufacturer's instructions. The detection limit for IL-6 was 5 pg/ml.

### Flow cytometry analysis of bone marrow cells

One femur was flushed with 2 ml of PBS through the bone cavity to harvest bone marrow cells. A Tris-buffered 0.83% NH_4_Cl solution, pH 7.29 was used to lyse erythrocytes, and the cells were then washed and re-suspended in fluorescence-activated cell sorting (FACS)-buffer (PBS supplemented with 1% fetal calf serum and 0.1% NaAz). Labelling of cell surface markers was performed using anti-CD19 PE, anti-CD3 APC, anti-CD4 PerCP, and anti-CD8 FITC antibodies (BD, Franklin Lakes, NJ, USA).

### Statistical analysis

The Kruskall-Wallis test followed by a *post hoc *test were used for comparisons between all groups in each experiment. A *P *value less than 0.05 was considered significant. The Graph Pad Prism 5 program was used for the statistical evaluations and calculations of area under the curve (AUC), according to the manufacturer's instructions.

## Results

### Treatment with Dex combined with E2 inhibits arthritis and prevents osteoporosis

OVX or sham-operated female DBA/1-mice were immunized for CIA in order to examine the anti-arthritic and anti-osteoporotic properties of Dex combined with E2. The mice were treated from the start of arthritis development (day 23 post immunization) with Dex (125 μg/day), E2 (1.0 μg/day), the combination of Dex + E2, or vehicle. Body weights were continuously followed and the maximum changes in weight ranged from -3.3% to +12.3%, calculated from the start of hormone treatment (day 23) until the termination of the experiment (day 44) (Sham/Veh + 5.4%; OVX/Veh + 6.8%; OVX/E2 + 12.3%; OVX/Dex -3.1%; OVX/Dex + E2 -3.3%).

**Figure 1:**
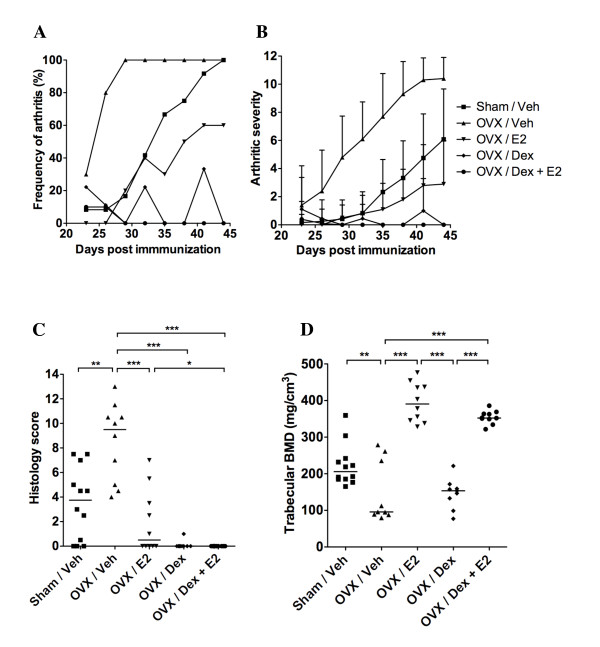
**Addition of 17β-estradiol to dexamethasone-treated arthritic mice protects against osteoporosis induced by ovariectomy and arthritis**. Female sham-operated (*n *= 12) or ovariectomy (OVX) mice treated with vehicle (Veh; *n *= 10), 17β-estradiol (E2; *n *= 10), dexamethasone (Dex; *n *= 9), or a combination of both Dex and E2 (*n *= 10) were used in order to evaluate the effects of treatment on arthritis and bone mineral density (BMD). Treatment was administered five days per week, starting after the first appearance of arthritis (day 23), and continued until the termination of the experiment (day 44). (**a**) Frequency and (**b**) severity of arthritis were evaluated every third day. Severity is expressed as the mean ± standard deviation in each group. (**c**) Histologic scores of destruction in paw sections. (**d**) Trabecular BMD of one femur. Scatter plots represent scores of individual mice, and bars show the median in each group. The non-parametric Kruskall-Wallis test with *post hoc *comparison was used for statistical evaluation of all parameters, *P<0.05, **P<0.01, ***P<0.001.

Arthritis scores of the paws were evaluated every third day. In the OVX vehicle control group, all mice had developed arthritis on day 29 after the first immunization (Figure 1a), whereas the debut was delayed in the sham-operated mice (100% frequency on day 44). Only 60% of the OVX mice treated with E2 developed arthritis. Treatment with Dex was highly protective and only 30% showed signs of disease. All mice received treatment for five days per week and clinical arthritis was seen at two time points in the Dex treatment group, when the mice had not been given Dex for two consecutive days. The symptoms quickly abated with continued treatment. The mice treated with the combination of both Dex and E2 did not display any signs of arthritis at any time points during the study. The frequency of arthritis differed significantly between the OVX control and the E2, Dex, and Dex + E2 groups from day 26 post immunization (*P *<0.01), and the differences were sustained throughout the treatment period. In addition, calculation of the AUC for the frequency of arthritis from day 23 to 44 (AUC_(day 23-44)_), showed that combined treatment with Dex and E2 resulted in a five-fold lower AUC_(day 23-44) _compared with Dex treatment alone. (AUC_(day 23-44)_: Sham/Veh = 1063, OVX/Veh = 1935, OVX/E2 = 690, OVX/Dex = 233, OVX/Dex + E2 = 45).

The severity of the arthritic disease was ameliorated in sham-operated mice, as well as in OVX mice treated with E2, compared with OVX controls (Figure 1b). In mice treated with Dex alone, mild signs of arthritis could be seen on two occasions when the mice had not been given treatment for two days, (corresponding to the peaks displayed in Figure 1a); however, mice receiving the combination of Dex + E2 did not display any signs of arthritis. OVX control mice had significantly higher arthritic severity score (*P*<0.01) from day 26 post immunization, compared with all other treatment groups (OVX/E2, OVX/Dex and OVX/Dex + E2).

Histologic examination of the paw sections revealed severe destruction of the joints from the vehicle-treated control mice (median destruction score of 9.5 in OVX vehicle mice) (Figure 1c). Sham-operated mice or OVX mice treated with E2 displayed significantly lower histology scores (median scores of 3.8 and 0.5, respecitvely). Dex treatment alone or in combination with E2 resulted in completely preserved joint structures, with median destruction scores of 0.

As expected, the trabecular BMD was significantly reduced in OVX mice compared with sham-operated controls (Figure 1d). Although the arthritic disease was successfully abated by treatment with Dex, the BMD was not affected by Dex treatment compared with OVX controls. However, combination of Dex and E2 treatment resulted in a maintained BMD to the same extent as E2 alone. In conclusion, these results show that combined treatment with glucocorticoids and estradiol successfully inhibits the development of arthritis and the accompanying joint destruction, and effectively preserves the BMD.

### Treatment with Dex combined with Ral inhibits arthritis and prevents osteoporosis

Since long-term treatment with estradiol is no longer recommended due to severe side effects, we wanted to examine if the combination of Ral with Dex treatment could be as beneficial in inhibiting arthritis and protecting the BMD as E2 + Dex in this model of arthritis. As described in the materials and methods section, OVX or sham-operated female DBA/1-mice were treated from the start of arthritis development (day 22 post immunization) with Dex (125 μg/day), Ral (120 μg/day), the combination of Dex + Ral, or controls. Body weights were continuously followed and the maximum changes in weight ranged from -5.5% to +3.7% calculated from the start of hormone treatment (day 22) until the termination of the experiment (day 49) (Sham/Veh -3.3%; OVX/Veh +3.7%; OVX/Ral +4.0%; OVX/Dex -5.5%; OVX/Dex + Ral -1.2%).

**Figure 2:**
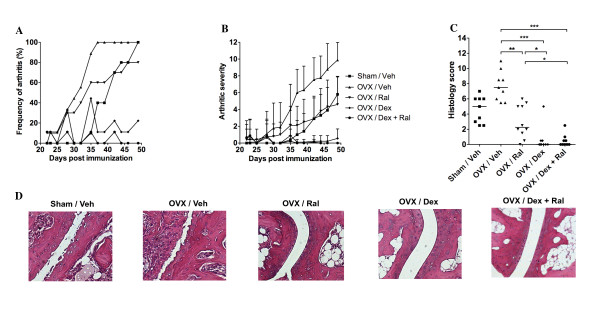
**Treatment with dexamethasone alone or in combination with raloxifene reduces arthritis and protects against joint destruction**. Female sham-operated (*n *= 10) or ovariectomy (OVX) mice treated with vehicle (Veh; *n *= 9), raloxifene (Ral; *n *= 10), dexamethasone (Dex; *n *= 9), or a combination of both Dex and Ral (*n *= 9) were used in order to evaluate the effects of treatment on arthritis and bone mineral density (BMD). Treatment was administered five days per week, starting after the first appearance of arthritis (day 22), and continued until the termination of the experiment (day 49). **(a) **Frequency and **(b) **severity were evaluated every two to three days. Severity is expressed as the mean ± standard deviation in each group. **(c) **Histologic destruction scores of paw sections. Scatter plots show the scores of individual mice, and bars show the median in each group. **(d) **Representative images of paw tissue sections, revealing the effects of treatment on histologic features in each group. The non-parametric Kruskall-Wallis test with *post hoc *comparison was used for statistical evaluation of all parameters, *P<0.05, **P<0.01, ***P<0.001.

Arthritis scores of the paws were evaluated every two to three days. In the OVX vehicle control group, all mice had developed arthritis on day 37 after the first immunization (Figure 2a), whereas in the group of sham-operated mice the arthritis debut was delayed (100% frequency on day 49). Of the Ral treated OVX mice, 80% developed arthritis. Treatment with Dex was highly protective, with a maximum of 40% ever showing signs of disease. Similar to the previous experimental setup (using Dex and E2), the mice treated with Dex alone or the combination of Dex + Ral showed minor flares of clinical arthritis at the time-points without treatment for two days. The frequency of arthritis differed significantly between the OVX control group and the Dex, and Dex + Ral groups from day 30 post immunization (*P*<0.05). The differences were sustained throughout the treatment period. In addition, calculation of the AUC for the frequency of arthritis during the days 22 to 49 (AUC_(day 22-49)_), showed that combined treatment with Dex and Ral resulted in a four-fold lower AUC_(day 22-49) _compared with Dex-treatment alone. (AUC_(day 22-49)_: Sham/Veh = 890, OVX/Veh = 1,878, OVX/Ral = 1,335, OVX/Dex = 406, OVX/Dex+Ral = 106).

As expected the severity of arthritis was ameliorated in the Sham/Veh and OVX/Ral groups compared with the OVX/Veh (Figure 2b). In mice treated with Dex alone or Dex + Ral, mild signs of arthritis could be seen occasionally, but the severity of arthritis differed significantly (*P*<0.05) for both groups as compared with OVX controls from day 30, and the difference was sustained throughout the treatment period. OVX control mice had significantly higher (*P*<0.01) arthritic severity scores from day 37 post immunization compared with the other treatment groups (OVX/Ral, OVX/Dex and OVX/Dex + Ral).

Severe destruction of the joints in the OVX control mice (median destruction score of 7.5) (Figure 2c to 2d) was revealed by histologic examination of the paws. OVX mice treated with Ral displayed significantly lower histology score (median score of 2.3). Dex-treatment alone or in combination with Ral resulted in completely preserved joint structure, with median destruction scores of 0. In Figure 2d, representative pictures of the H&E stained paw sections from each treatment group are presented.

**Figure 3:**
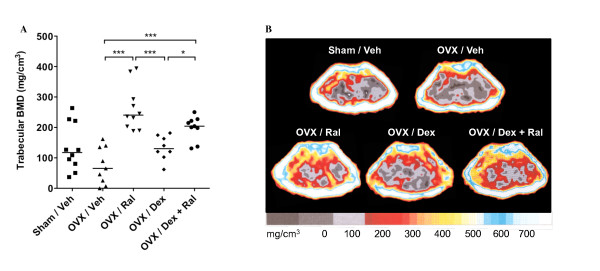
**The combination of raloxifene with dexamethasone-treatment preserves the bone from osteoporosis induced by arthritis and ovariectomy**. Female sham-operated (*n *= 10) or ovariectomy (OVX) mice treated with vehicle (Veh; *n *= 9), raloxifene (Ral; *n *= 10), dexamethasone (Dex; *n *= 8), or a combination of both Dex and Ral (*n *= 9) were used in order to evaluate the effects on trabecular bone mineral density (BMD). Treatment was administered five days per week, starting after the first appearance of arthritis (day 22), and continued until the termination of the experiment (day 49). (**a) **Scatter plots of individual data show the trabecular BMD of one femur and bars indicate the median per group. Non-parametric Kruskall-Wallis test with *post hoc *comparison was used for statistical evaluation of all parameters, *P<0.05, **P<0.01, ***P<0.001. **(b) **Representative peripheral quantitative computer tomography (pQCT) images of cross-sections of the femur showing the BMD. The scale beneath indicates the density of the bone, from 0 (gray) to >750 mg/cm^3 ^(white).

The trabecular BMD was significantly increased after treatment with Ral, compared with OVX control mice (Figure 3a), and the combination of Dex + Ral treatment resulted in a maintained BMD to the same extent as treatment with Ral alone. In Figure 3b representative pictures of the pQCT scans from each treatment group are shown. In conclusion, treatment with the combination of glucocorticoids and Ral totally abrogates the development of arthritis, joint destruction, and trabecular bone loss.

### Serological markers of cartilage destruction, bone destruction, and inflammation decrease after treatment with the combination of Dex and Ral

**Figure 4:**
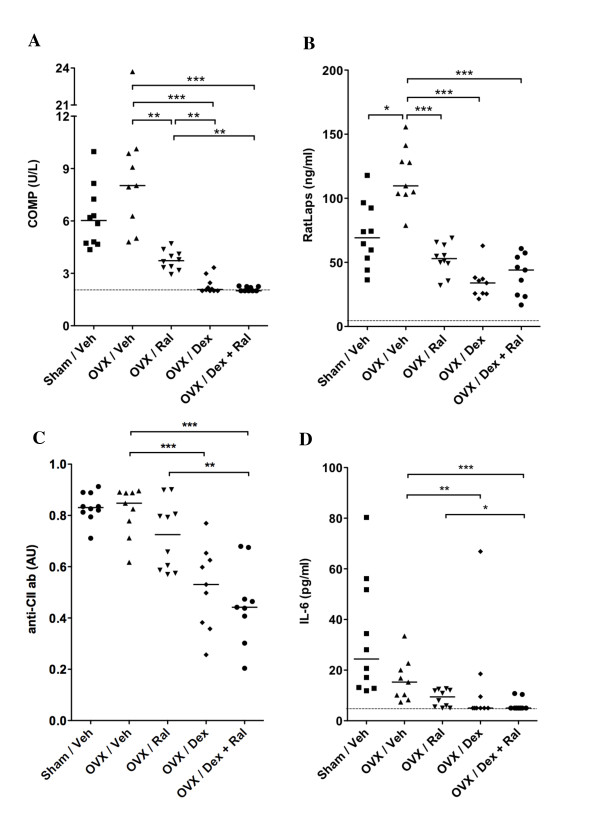
**Treatment with dexamethasone alone or in combination with raloxifene reduces the serum levels of COMP, RatLaps and inflammatory markers in arthritic mice**. Female sham-operated (*n *= 10) or ovariectomy (OVX) mice treated with vehicle (Veh; *n *= 9), raloxifene (Ral; *n *= 10), dexamethasone (Dex; *n *= 9), or a combination of both Dex and Ral (*n *= 9) were used. Treatment was administered five days per week, started after the first appearance of arthritis (day 22) and continued until the termination of the experiment (day 49). The effects of the different treatments on cartilage destruction, bone destruction and inflammation were investigated. Serum levels of (**a**) COMP (cartilage destruction), (**b**) RatLaps (bone destruction), and **(c) **anti-collagen type II (CII) antibodies were measured by ELISA. Serum levels of (**d**) IL-6 were measured by cytometric bead assay. The detection limit for COMP was 2 U/L, for RatLaps 6 ng/ml, and for IL-6 5 pg/ml. The levels of anti-CII antibodies are described in arbitrary units (AU). Scatter plots of individual data are presented and bars indicate the median. The non-parametric Kruskall-Wallis test with *post hoc *comparison was used for statistical evaluation of all parameters, *P<0.05, **P<0.01, ***P<0.001.

In CIA, the levels of COMP are elevated in serum due to increased destruction of joint cartilage. Treatment with Dex alone or the combination of Dex + Ral completely inhibited any detection of COMP in serum of these mice (Figure 4a). In order to measure bone resorption, collagen type I cross-links (RatLaps) were analyzed in serum (Figure 4b). As expected, the levels of RatLaps were significantly decreased in mice treated with Ral, Dex, or the combination of Dex + Ral, compared with OVX controls. An increased level of anti-CII antibodies in serum is a marker for an ongoing specific immune-activation towards CII. Treatment with Dex or the combination of Dex + Ral resulted in significantly lower levels of IgG anti-CII antibodies in serum (Figure 4c). An increase in the serum levels of IL-6 in CIA is a manifestation of the general inflammatory disease. Mice in the OVX control group displayed the most severe disease, and as expected serum levels of IL-6 were increased in this group compared with the mice treated with Dex or the combination of Dex + Ral (Figure 4d).

### Dex-treatment reduces the levels of B cells, but not T cells, in the bone marrow

**Figure 5:**
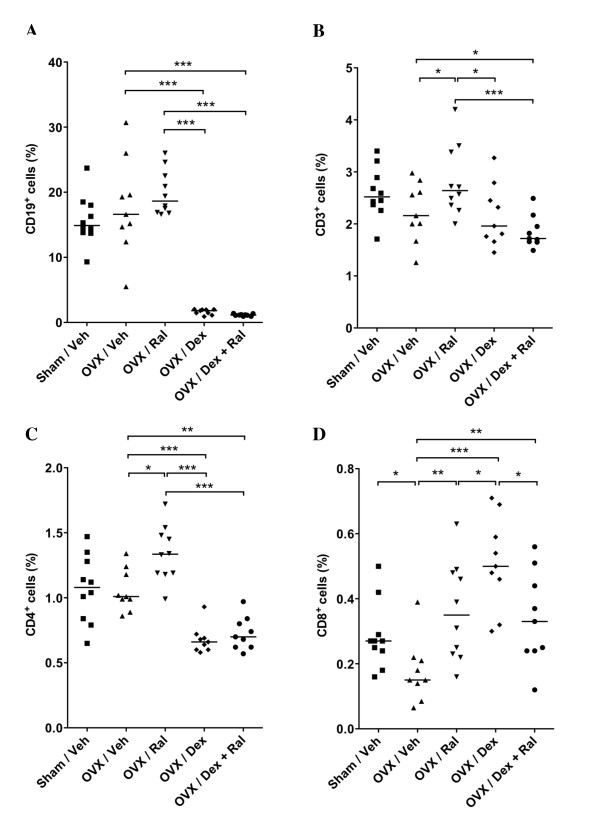
**Treatment with dexamethasone alone or in combination with raloxifene reduces the B cell frequency in bone marrow of arthritic mice**. Female sham-operated (*n *= 10) or ovariectomy (OVX) mice treated with vehicle (Veh; *n *= 9), raloxifene (Ral; *n *= 10), dexamethasone (Dex; *n *= 9), or a combination of both Dex and Ral (*n *= 9) were used. Treatment was administered five days per week, started after the first appearance of arthritis (day 22) and continued until the termination of the experiment (day 49). Flow cytometry analysis of bone marrow cells was performed in order to investigate the effects of treatment on different lymphocyte phenotypes. Bone marrow cells from one femur were harvested and labelled with antibodies against (**a**) CD19, **(b) **CD3, **(c) **CD4, or **(d) **CD8. Scatter plots of individual data are presented and bars indicate the median in each group. The non-parametric Kruskall-Wallis test with *post hoc *comparison was used for statistical evaluation of all parameters, *P<0.05, **P<0.01, ***P<0.001.

Treatment with corticosteroids are known to induce apoptosis of B lineage precursors in the bone marrow, as well as of developing T cells in the thymus [17,18]. Flow cytometry analysis was performed in order to investigate the effects of treatment on bone marrow lymphocytes. As expected, treatment with Dex or Dex + Ral resulted in a substantial depletion of CD19 positive B cells in the bone marrow (Figure 5a). A tendency towards lower levels of CD3 positive T cells was detected after treatment with Dex or Dex + Ral compared with OVX controls (Figure 5b). However, when CD4 and CD8 positive T cells were studied separately, decreased levels of CD4 positive cells could be detected in the Dex or Dex + Ral groups compared with the OVX control group (Figure 5c), while CD8 positive T cells were increased (Figure 5d).

## Discussion

Estrogens can influence the pathogenesis of many inflammatory diseases including RA [19]. The beneficial effects of E2 on the development of murine experimental arthritis, and on osteoporosis associated with arthritis and ovariectomy, have been previously well documented [14,20,21]. Nevertheless, long-term use of estrogen in humans is associated with an increased risk of breast cancer and thrombosis, and is therefore no longer recommended [22,23]. The SERM Ral is approved for treatment of patients with postmenopausal osteoporosis, and is also approved as prophylaxis for invasive breast cancer in the USA. However, studies have shown that treatment with Ral increases the risk of deep venous thrombosis and pulmonary embolism [24]. We recently investigated if Ral was as beneficial as E2 on arthritis-development and inflammation-induced bone loss. Indeed, our studies revealed that Ral reduces the frequency and severity of CIA, and preserves the bone [11,12]. Those results were successfully repeated in this study.

Glucocorticoids are potent anti-inflammatory and immunosuppressive agents that are widely used to treat both acute and chronic inflammation, such as RA. However, long-term treatment with glucocorticoids in humans is associated with severe side effects including metabolic disease, cardiovascular disease, avascular necrosis, and osteoporosis [25-28]. The aim of the current study was to investigate if addition of Ral or E2 to ovariectomized Dex-treated arthritic mice could prevent bone loss while simultaneously ameliorating the arthritic disease. In clinical trials, the addition of estrogen replacement therapy has been shown to have positive effects on arthritis-associated and glucocorticoid-induced bone loss [8,29,30]. In addition, we have previously shown that treatment with the combination of Dex + E2 have additive suppressive effects on the T cell-mediated delayed type hypersensitivity reaction in mice [31]. However, the effects of combined treatment with Ral and Dex on ovariectomized mice with inflammation-induced osteoporosis have not previously been investigated.

In this study we show that treatment with Dex alone significantly reduced the frequency and severity of arthritis as well as the histologic evaluation scores of the joints compared with control treated mice. The combination of Dex + Ral or Dex + E2 completely abrogated any signs of the disease and the median histologic joint destruction score was kept at 0. Previous studies have demonstrated that mice are susceptible to glucocorticoid-induced osteoporosis [32,33]. Interestingly, in this experimental setup we did not find any differences in trabecular BMD between the OVX/Veh and the OVX/Dex group. This could be due to the fact that all mice were ovariectomized four weeks prior to the treatment start, and therefore likely already had low BMD at the start of the treatment. In spite of this, the combined treatment with Ral + Dex or E2 + Dex resulted in preserved BMD at levels similar to the groups that received treatment with Ral or E2 alone. COMP and RatLaps are markers for cartilage destruction and bone destruction, respectively. Mice treated with Dex alone or Dex + Ral displayed low or undetectable levels of COMP and RatLaps compared with controls, reflecting the low levels of joint and bone destruction in these groups.

The mechanisms for the anti-inflammatory effects of glucocorticoids involve inhibition of vascular permeability that occurs as an inflammatory response, as well as decreased leukocyte migration into inflamed sites [34]. The effects of estrogens on inflammation is a still unresolved paradox where estrogens can have both anti-inflammatory and pro-inflammatory roles depending on, for example, the immune stimulus, the concentration of the estrogen, the target organ, the specific disease, and the cell types involved [19]. The beneficial effects of estrogen on arthritis are well documented [14,20,21]; however, the mechanisms are largely unknown. The levels of anti-CII antibodies and IL-6 can be used as markers for specific and generalized inflammation in CIA. In this study the levels of anti-CII antibodies were significantly decreased in mice treated with Dex alone or with the combination of Dex + Ral compared with controls. In addition, IL-6 was undetectable in the majority of mice treated with Dex or Dex + Ral.

Glucocorticoids have pro-apoptotic effects on early progenitor B cells in the bone marrow [18,35], as well as on immature double positive T cells in the thymus [17,35]. In contrast, mature single positive thymocytes and peripheral T cells are less sensitive to glucocorticoid-induced apoptosis [35]. We have previously shown that treatment with E2 or Ral reduces the number of double positive T cells in the thymus as well as the frequency of B cells in the bone marrow [36,37]. As expected, in this study Dex or Dex + Ral treatment resulted in a significant reduction of CD19+ bone marrow cells compared with controls (Figure 5a), while there was only a tendency towards a reduction of CD3+ bone marrow cells (Figure 5b). Interestingly, when the CD3+ bone marrow cells were divided into CD4+ and CD8+ T cells, Dex or Dex + Ral treatment resulted in a decrease in CD4+ T cells and an increase in CD8+ T cells compared with controls. The reason for this discrepancy is unknown; however, we speculate that mature CD4+ T cells are sensitive to glucocorticoid-induced apoptosis while CD8+ T cells are not.

The mechanisms behind the ameliorating effects of E2 or Ral on arthritis, and their anabolic effects on the skeleton, are to a large extent unknown. We have previously shown that signalling via estrogen receptor (ER) α, but not ERβ or GPR30, protects against ovariectomy-induced trabecular bone loss and ameliorates CIA [38-41]. Ral has been shown to bind with high affinity to ERα and functions as an estrogen agonist in bone and on serum lipids, but acts as an antagonist in uterus and breast tissue [42-46]. It is therefore reasonable to believe that the protective effect on bone and the arthritic disease after treatment with the combination of Dex and Ral is due to the stimulating effect of Ral on ERα.

Bisphosphonates reduce bone turnover by altering osteoclast activity and are often considered first-line therapy for glucocorticoid-induced and inflammation-induced osteoporosis [47]. Given the results in this study, we speculate that combined treatment with glucocorticoids and Ral in postmenopausal RA patients might be an excellent alternative to treatment with glucocorticoids and bisphosphonates. The next step will be to perform clinical studies in order to reveal the effects of treatment with a combination of glucocorticoids and Ral in postmenopausal RA patients.

Numerous new SERMs are currently undergoing clinical development for the prevention and/or treatment of postmenopausal osteoporosis [48]. For example, lasofoxifene was recently described to be associated with reduced risks of fractures, ER-positive breast cancer, coronary heart disease, and stroke in postmenopausal patients with osteoporosis [49]. In addition, bazedoxifene was shown to significantly reduce the risk of fractures in postmenopausal women with osteoporosis, as well as prevent bone loss and reduce bone turnover equally well as Ral [50,51]. If these new SERMs have similar anti-arthritic effects as Ral, and if the combination with glucocorticoids has similar effects as we have shown in this study, remains to be investigated.

## Conclusions

Results from this study show that addition of Ral or estradiol to Dex treatment in arthritic mice prevents generalized bone loss and development of arthritis. We suggest that combined treatment with glucocorticoids and Ral or other SERMs can be beneficial in order to ameliorate the arthritic disease and simultaneously preserve the bone in postmenopausal patients with RA.

## Abbreviations

AUC: area under the curve; BMD: bone mineral density; BSA: bovine serum albumin; CIA: collagen-induced arthritis; CII: type II collagen; COMP: cartilage oligomeric matrix protein; Dex: dexamethasone; E2: 17β-estradiol; ELISA: enzyme-linked immunosorbent assay; ER: estrogen receptor; FACS: fluorescence-activated cell sorting; H&E: hematoxylin and eosin; HRT: hormone replacement therapy; IL: interleukin; ip: intraperitoneal; OVX: ovariectomy; PBS: phosphate buffered saline; pQCT: peripheral quantitative computed tomography; RA: rheumatoid arthritis; Ral: raloxifene; sc: subcutaneous; SERM: selective estrogen receptor modulator.

## Competing interests

The authors declare that they have no competing interests.

## Authors' contributions

HC and CO participated in study design, interpretation of data and manuscript preparation. MKL aided with analysis of data. AK and AA aided with acquisition of data. The study was performed mainly by UI and CJ. All authors read and approved the final manuscript.

## References

[B1] Kvien TK Glennas A Knudsrod OG Smedstad LM Mowinckel P Forre O The prevalence and severity of rheumatoid arthritis in Oslo. Results from a county register and a population survey Scand J Rheumatol 1997 26 6 412 418 10.3109/03009749709065712 9433400

[B2] Kvien TK Uhlig T Odegard S Heiberg MS Epidemiological aspects of rheumatoid arthritis: the sex ratio Ann N Y Acad Sci 2006 1069 212 222 10.1196/annals.1351.019 16855148

[B3] Goemaere S Ackerman C Goethals K De Keyser F Van der Straeten C Verbruggen G Mielants H Veys EM Onset of symptoms of rheumatoid arthritis in relation to age, sex and menopausal transition J Rheumatol 1990 17 1620 1622 2084234

[B4] Forsblad-D'Elia H Larsen A Waltbrand E Kvist G Mellstrom D Saxne T Ohlsson C Nordborg E Carlsten H Radiographic joint destruction in postmenopausal rheumatoid arthritis is strongly associated with generalised osteoporosis Ann Rheum Dis 2003 62 617 623 10.1136/ard.62.7.617 12810422 PMC1754591

[B5] Sinigaglia L Nervetti A Mela Q Bianchi G Del Puente A Di Munno O Frediani B Cantatore F Pellerito R Bartolone S La Montagna G Adami S A multicenter cross sectional study on bone mineral density in rheumatoid arthritis. Italian Study Group on Bone Mass in Rheumatoid Arthritis J Rheumatol 2000 27 2582 2589 11093437

[B6] Chantler IW Davie MW Evans SF Rees JS Oral corticosteroid prescribing in women over 50, use of fracture prevention therapy, and bone densitometry service Ann Rheum Dis 2003 62 350 352 10.1136/ard.62.4.350 12634236 PMC1754497

[B7] Lafage-Proust MH Boudignon B Thomas T Glucocorticoid-induced osteoporosis: pathophysiological data and recent treatments Joint Bone Spine 2003 70 109 118 10.1016/S1297-319X(03)00016-2 12713854

[B8] Forsblad-D'Elia H Larsen A Mattsson LA Waltbrand E Kvist G Mellstrom D Saxne T Ohlsson C Nordborg E Carlsten H Influence of hormone replacement therapy on disease progression and bone mineral density in rheumatoid arthritis J Rheumatol 2003 30 1456 1463 12858441

[B9] Stefanick ML Estrogens and progestins: background and history, trends in use, and guidelines and regimens approved by the US Food and Drug Administration Am J Med 2005 118 Suppl 12B 64 73 16414329 10.1016/j.amjmed.2005.09.059

[B10] Temin S American Society of Clinical Oncology clinical practice guideline update on the use of pharmacologic interventions including tamoxifen, raloxifene, and aromatase inhibition for breast cancer risk reduction Gynecol Oncol 2009 115 1 132 134 10.1016/j.ygyno.2009.06.006 19716939 PMC4119170

[B11] Jochems C Islander U Kallkopf A Lagerquist M Ohlsson C Carlsten H Role of raloxifene as a potent inhibitor of experimental postmenopausal polyarthritis and osteoporosis Arthritis Rheum 2007 56 10 3261 3270 10.1002/art.22873 17907171

[B12] Jochems C Lagerquist M Hakansson C Ohlsson C Carlsten H Long-term anti-arthritic and anti-osteoporotic effects of raloxifene in established experimental postmenopausal polyarthritis Clin Exp Immunol 2008 152 593 597 10.1111/j.1365-2249.2008.03660.x 18435803 PMC2453214

[B13] Holmdahl R Bockermann R Backlund J Yamada H The molecular pathogenesis of collagen-induced arthritis in mice--a model for rheumatoid arthritis Ageing Res Rev 2002 1 135 147 10.1016/S0047-6374(01)00371-2 12039453

[B14] Jochems C Islander U Erlandsson M Verdrengh M Ohlsson C Carlsten H Osteoporosis in experimental postmenopausal polyarthritis: the relative contributions of estrogen deficiency and inflammation Arthritis Res Ther 2005 7 R837 843 10.1186/ar1753 15987485 PMC1175035

[B15] Holmdahl R Jansson L Larsson E Rubin K Klareskog L Homologous type II collagen induces chronic and progressive arthritis in mice Arthritis Rheum 1986 29 106 113 10.1002/art.1780290114 3947407

[B16] Windahl SH Vidal O Andersson G Gustafsson JA Ohlsson C Increased cortical bone mineral content but unchanged trabecular bone mineral density in female ERbeta(-/-) mice J Clin Invest 1999 104 895 901 10.1172/JCI6730 10510330 PMC408552

[B17] Ashwell JD Lu FW Vacchio MS Glucocorticoids in T cell development and function* Annual Review of Immunology 2000 18 309 345 10.1146/annurev.immunol.18.1.309 10837061

[B18] Igarashi H Medina KL Yokota T Rossi MI Sakaguchi N Comp PC Kincade PW Early lymphoid progenitors in mouse and man are highly sensitive to glucocorticoids Int Immunol 2005 17 501 511 10.1093/intimm/dxh230 15746243

[B19] Straub RH The complex role of estrogens in inflammation Endocr Rev 2007 28 521 574 10.1210/er.2007-0001 17640948

[B20] Holmdahl R Jansson L Andersson M Female sex hormones suppress development of collagen-induced arthritis in mice Arthritis Rheum 1986 29 1501 1509 10.1002/art.1780291212 3801072

[B21] Yamasaki D Enokida M Okano T Hagino H Teshima R Effects of ovariectomy and estrogen replacement therapy on arthritis and bone mineral density in rats with collagen-induced arthritis Bone 2001 28 634 640 10.1016/S8756-3282(01)00426-4 11425652

[B22] Anderson GL Limacher M Assaf AR Bassford T Beresford SA Black H Bonds D Brunner R Brzyski R Caan B Chlebowski R Curb D Gass M Hays J Heiss G Hendrix S Howard BV Hsia J Hubbell A Jackson R Johnson KC Judd H Kotchen JM Kuller L LaCroix AZ Lane D Langer RD Lasser N Lewis CE Manson J Effects of conjugated equine estrogen in postmenopausal women with hysterectomy: the Women's Health Initiative randomized controlled trial JAMA 2004 291 1701 1712 15082697 10.1001/jama.291.14.1701

[B23] Rossouw JE Anderson GL Prentice RL LaCroix AZ Kooperberg C Stefanick ML Jackson RD Beresford SA Howard BV Johnson KC Kotchen JM Ockene J Risks and benefits of estrogen plus progestin in healthy postmenopausal women: principal results From the Women's Health Initiative randomized controlled trial JAMA 2002 288 321 333 10.1001/jama.288.3.321 12117397

[B24] Adomaityte J Farooq M Qayyum R Effect of raloxifene therapy on venous thromboembolism in postmenopausal women. A meta-analysis Thromb Haemost 2008 99 2 338 342 18278183

[B25] Bauer M Thabault P Estok D Christiansen C Platt R Low-dose corticosteroids and avascular necrosis of the hip and knee Pharmacoepidemiol Drug Saf 2000 9 187 191 10.1002/1099-1557(200005/06)9:3<187::AID-PDS494>3.0.CO;2-J 19025819

[B26] de Vries F Pouwels S Lammers JW Leufkens HG Bracke M Cooper C van Staa TP Use of inhaled and oral glucocorticoids, severity of inflammatory disease and risk of hip/femur fracture: a population-based case-control study J Intern Med 2007 261 170 177 17241182 10.1111/j.1365-2796.2006.01754.x

[B27] Vegiopoulos A Herzig S Glucocorticoids, metabolism and metabolic diseases Mol Cell Endocrinol 2007 275 43 61 10.1016/j.mce.2007.05.015 17624658

[B28] Wei L MacDonald TM Walker BR Taking glucocorticoids by prescription is associated with subsequent cardiovascular disease Ann Intern Med 2004 141 764 770 15545676 10.7326/0003-4819-141-10-200411160-00007

[B29] Hall GM Daniels M Doyle DV Spector TD Effect of hormone replacement therapy on bone mass in rheumatoid arthritis patients treated with and without steroids Arthritis Rheum 1994 37 1499 1505 10.1002/art.1780371014 7945476

[B30] Lukert BP Johnson BE Robinson RG Estrogen and progesterone replacement therapy reduces glucocorticoid-induced bone loss J Bone Miner Res 1992 7 1063 1069 1329440 10.1002/jbmr.5650070909

[B31] Carlsten H Verdrengh M Taube M Additive effects of suboptimal doses of estrogen and cortisone on the suppression of T lymphocyte dependent inflammatory responses in mice Inflamm Res 1996 45 26 30 10.1007/BF02263501 8821775

[B32] Weinstein RS Glucocorticoid-induced osteoporosis Rev Endocr Metab Disord 2001 2 65 73 10.1023/A:1010007108155 11708295

[B33] Weinstein RS Jilka RL Parfitt AM Manolagas SC Inhibition of osteoblastogenesis and promotion of apoptosis of osteoblasts and osteocytes by glucocorticoids. Potential mechanisms of their deleterious effects on bone J Clin Invest 1998 102 274 282 10.1172/JCI2799 9664068 PMC508885

[B34] Coutinho AE Chapman KE The anti-inflammatory and immunosuppressive effects of glucocorticoids, recent developments and mechanistic insights Mol Cell Endocrinol 2011 335 2 13 10.1016/j.mce.2010.04.005 20398732 PMC3047790

[B35] Baschant U Tuckermann J The role of the glucocorticoid receptor in inflammation and immunity J Steroid Biochem Mol Biol 2010 120 69 75 10.1016/j.jsbmb.2010.03.058 20346397

[B36] Erlandsson MC Gomori E Taube M Carlsten H Effects of raloxifene, a selective estrogen receptor modulator, on thymus, T cell reactivity, and inflammation in mice Cell Immunol 2000 205 103 109 10.1006/cimm.2000.1719 11104582

[B37] Erlandsson MC Jonsson CA Lindberg MK Ohlsson C Carlsten H Raloxifene-and estradiol-mediated effects on uterus, bone and B lymphocytes in mice J Endocrinol 2002 175 319 327 10.1677/joe.0.1750319 12429030

[B38] Engdahl C Jochems C Windahl SH Borjesson AE Ohlsson C Carlsten H Lagerquist MK Amelioration of collagen-induced arthritis and immune-associated bone loss through signaling via estrogen receptor alpha, and not estrogen receptor beta or G protein-coupled receptor 30 Arthritis Rheum 2010 62 524 533 20112355 10.1002/art.25055

[B39] Lindberg MK Moverare S Skrtic S Alatalo S Halleen J Mohan S Gustafsson JA Ohlsson C Two different pathways for the maintenance of trabecular bone in adult male mice J Bone Miner Res 2002 17 555 562 10.1359/jbmr.2002.17.4.555 11918213

[B40] Lindberg MK Weihua Z Andersson N Moverare S Gao H Vidal O Erlandsson M Windahl S Andersson G Lubahn DB Carlsten H Dahlman-Wright K Gustafsson JA Ohlsson C Estrogen receptor specificity for the effects of estrogen in ovariectomized mice J Endocrinol 2002 174 167 178 10.1677/joe.0.1740167 12176656

[B41] Windahl SH Andersson N Chagin AS Martensson UE Carlsten H Olde B Swanson C Moverare-Skrtic S Savendahl L Lagerquist MK Leeb-Lundberg LM Ohlsson C The role of the G protein-coupled receptor GPR30 in the effects of estrogen in ovariectomized mice Am J Physiol Endocrinol Metab 2009 296 E490 496 19088255 10.1152/ajpendo.90691.2008

[B42] Cummings SR Eckert S Krueger KA Grady D Powles TJ Cauley JA Norton L Nickelsen T Bjarnason NH Morrow M Lippman ME Black D Glusman JE Costa A Jordan VC The effect of raloxifene on risk of breast cancer in postmenopausal women: results from the MORE randomized trial. Multiple Outcomes of Raloxifene Evaluation JAMA 1999 281 2189 2197 10.1001/jama.281.23.2189 10376571

[B43] Ettinger B Black DM Mitlak BH Knickerbocker RK Nickelsen T Genant HK Christiansen C Delmas PD Zanchetta JR Stakkestad J Gluer CC Krueger K Cohen FJ Eckert S Ensrud KE Avioli LV Lips P Cummings SR Reduction of vertebral fracture risk in postmenopausal women with osteoporosis treated with raloxifene: results from a 3-year randomized clinical trial. Multiple Outcomes of Raloxifene Evaluation (MORE) Investigators JAMA 1999 282 637 645 10.1001/jama.282.7.637 10517716

[B44] Li X Takahashi M Kushida K Inoue T The preventive and interventional effects of raloxifene analog (LY117018 HCL) on osteopenia in ovariectomized rats J Bone Miner Res 1998 13 1005 1010 10.1359/jbmr.1998.13.6.1005 9626632

[B45] Sato M Kim J Short LL Slemenda CW Bryant HU Longitudinal and cross-sectional analysis of raloxifene effects on tibiae from ovariectomized aged rats J Pharmacol Exp Ther 1995 272 1252 1259 7891341

[B46] Sato M Rippy MK Bryant HU Raloxifene, tamoxifen, nafoxidine, or estrogen effects on reproductive and nonreproductive tissues in ovariectomized rats Faseb J 1996 10 905 912 8666168 10.1096/fasebj.10.8.8666168

[B47] Adler RA Glucocorticoid-induced osteoporosis: management update Curr Osteoporos Rep 2010 8 10 14 10.1007/s11914-010-0003-6 20425085

[B48] Miller PD Derman RJ What is the best balance of benefits and risks among anti-resorptive therapies for postmenopausal osteoporosis? Osteoporos Int 2010 21 1793 1802 10.1007/s00198-010-1208-3 20309524

[B49] Cummings SR Ensrud K Delmas PD LaCroix AZ Vukicevic S Reid DM Goldstein S Sriram U Lee A Thompson J Armstrong RA Thompson DD Powles T Zanchetta J Kendler D Neven P Eastell R Lasofoxifene in postmenopausal women with osteoporosis N Engl J Med 2010 362 686 696 10.1056/NEJMoa0808692 20181970

[B50] Miller PD Chines AA Christiansen C Hoeck HC Kendler DL Lewiecki EM Woodson G Levine AB Constantine G Delmas PD Effects of bazedoxifene on BMD and bone turnover in postmenopausal women: 2-yr results of a randomized, double-blind, placebo-, and active-controlled study J Bone Miner Res 2008 23 525 535 18072873 10.1359/jbmr.071206

[B51] Silverman SL Christiansen C Genant HK Vukicevic S Zanchetta JR de Villiers TJ Constantine GD Chines AA Efficacy of bazedoxifene in reducing new vertebral fracture risk in postmenopausal women with osteoporosis: results from a 3-year, randomized, placebo-, and active-controlled clinical trial J Bone Miner Res 2008 23 1923 1934 10.1359/jbmr.080710 18665787

